# Role of Abandoned and Vacant Houses on *Aedes aegypti* Productivity

**DOI:** 10.4269/ajtmh.20-0829

**Published:** 2020-10-05

**Authors:** Roberto Barrera, Veronica Acevedo, Manuel Amador

**Affiliations:** Entomology and Ecology Team, Dengue Branch, CDC, San Juan, Puerto Rico

## Abstract

The control of container *Aedes* species by house inspections usually achieves insufficient coverage and impact because a percentage of residents are absent and some residents refuse inspections and treatments. In addition, another fraction of the buildings may be uninhabited, such as those for rent or sale, or abandoned. Although the productivity of *Aedes aegypti* has been investigated in abandoned lots, less is known about the importance of uninhabited buildings. We investigated *Ae. aegypti* pupal productivity in inhabited, vacant, and abandoned houses and its interaction with socioeconomic levels (SELs). We found pupae in containers of 386 houses (66 abandoned, 62 vacant, and 258 inhabited) in 19 neighborhoods in southern Puerto Rico from May to August 2017. Using a generalized linear model, we found a significant interaction between the status of the house (abandoned, vacant, and inhabited) and SELs (low, medium) on *Ae. aegypti* pupal abundance. More pupae were found in abandoned and inhabited houses of low SELs. The lowest productivity was found in vacant houses, regardless of the SEL. Most containers producing *Ae. aegypti* in low-SEL houses were discarded on backyards, whereas in medium SELs, most productivity came from containers in use. Septic tanks producing *Ae. aegypti* were found only in houses of low SELs, where most emerging mosquitoes came from inhabited houses. We did not find any pupae of *Ae. aegypti* on roofs. These results indicate that proper yard management could significantly reduce the production of *Ae. aegypti* and the risk of dengue infections in low-SEL neighborhoods.

## INTRODUCTION

Dengue cases and infected countries have been increasing in the last decades, threatening to expand into temperate areas.^1,2^ In addition, the arrival of new viruses, like chikungunya and Zika, and the concern for the re-emergence of outbreaks caused by the yellow fever virus demand improved control of *Aedes aegypti*, the main vector of these viruses. Effective *Ae. aegypti* and arboviral disease control must be carried out by a combination of tools to manage the immature and adult stages of this mosquito.^3^ One of the limitations of mosquito control involving house-to-house inspections is the possible important effect that some out-of-reach buildings (vacant, abandoned, or rejecting participation) might have on *Ae. aegypti* productivity, a topic that is not well understood. Poor vector control coverage, leading to sparse vector population mortality, results in ineffective control in urban areas with suboptimal household participation or accessibility.^3,4^

Integrated *Ae. aegypti* management programs must include multi-sectorial participation to address social and environmental limitations that contribute to mosquito proliferation.^5^ Deterioration of social or economic situations and natural disasters can produce exodus of residents,^6^ leaving many properties uninhabited. According to the U.S. Census, from 2010 to 2017 Puerto Rico had a decrease of 388,980 people, which resulted in 20.8% of houses being uninhabited.^7^ Abandoned lots and other public areas produce *Ae. aegypti* mosquitoes in a variety of containers that collect water.^8,9^ However, limited information is available about the role of uninhabited houses in *Ae. aegypti* productivity. A previous study reported that vacant houses and abandoned buildings (17.9%) in Salinas, Puerto Rico, produced 19.7% of *Ae. aegypti* pupae in contrast to 78.9% produced in inhabited houses and 1.4% in commercial or public buildings.^10^

Socioeconomic levels (SELs) should be considered to understand the contribution of uninhabited houses to the population of *Ae. aegypti*. For example, lower income urban areas tend to have higher mosquito densities.^11–13^ Lack of reliable services of piped water, domestic garbage collection, and knowledge about where *Ae. aegypti* are produced have been identified as causes for elevated mosquito densities.^11,12,14,15^ If uninhabited buildings and houses do not receive proper maintenance, they may harbor containers producing *Ae. aegypti*, the extent of which is largely unknown. The main goal of this study was to compare the relative contribution of uninhabited (vacant and abandoned) and inhabited properties to mosquito productivity in neighborhoods with contrasting SELs in Puerto Rico and to identify the main types of containers producing *Ae. aegypti* mosquitoes.

## METHODS

We compared the number of *Ae. aegypti* pupae found in containers of abandoned, vacant, and inhabited houses in 19 communities in Salinas, Arroyo, and Patillas municipalities, southern Puerto Rico, from May to August 2017 (Supplemental Table S1, Figure S1). The study communities were selected based on our perception of how neighborhoods of low and medium SELs looked like in Puerto Rico, although for analytic purposes, we assigned houses to each SEL based on a cluster analysis as described in the following texts. Although we originally planned to study pupal productivity (pupae/house, pupal/container type) in houses from a wider variation in SELs, we could not sample houses in high SELs because we had not yet started sampling in these neighborhoods when hurricanes Irma and Maria devastated Puerto Rico in September 2017. We visited each community in search of abandoned and vacant houses that could be accessed. For each abandoned or vacant house, we visited two adjacent inhabited houses for comparison purposes. Only inhabited houses next to inspected abandoned or vacant houses were included in the analysis. Sampling proceeded until we were able to inspect a minimum of 30 abandoned houses and 60 paired inhabited houses and 30 vacant houses and their corresponding 60 paired inhabited houses per SEL. Abandoned and vacant houses were dispersed within communities so that they did not represent aggregations or clusters.

We did not access the interior of houses in search of containers with water because we have observed in the past that they are not common in Puerto Rico. Abandoned houses had no maintenance or utilities, whereas vacant houses were mainly for rent or for sale and received some type of maintenance. We requested oral consent from an adult resident to enter and inspect the properties searching for containers producing mosquitoes. Access to abandoned and vacant houses was limited because we needed to find the property’s owner or caretaker to request access. Because we did not register personal identifiable information nor took human specimens, this study did not require institutional review board oversight. We used meteorological data from seven meteorological stations in or close to the studied neighborhoods (HOBO RX3000 remote data loggers, Onset Computer Corporation, Bourne, MA): four stations were in Salinas, one in Guayama, one in Patillas, and one in Arroyo municipality (Supplemental Figure S2). We calculated accumulated rainfall and average daily temperature and relative humidity for 7 days preceding each mosquito sampling date.

Community-level or average attributes of the sampled houses were 1) origin or development of the neighborhoods (private sector, land provided by the municipality, and invasion) that was provided by municipal authorities, and 2) the following average attributes obtained from census tracts (https://data.census.gov/cedsci/): property value, median income, average family size, percentage of residents without complete high school education, percentage of residents older than 60 years, percentage of uninhabited houses, and percentage of houses older than 30 years. We could not get information on the proportion of uninhabited houses that were abandoned or vacant. Household-level attributes were average lot size and House Condition Index.^16^ Average lot size was calculated by means of a geographical information system (ArcView 10, Esri, Redlands, CA) from a layer of georeferenced polygons representing lot sizes (Property Tax Office of Puerto Rico). We evaluated the yard and house conditions based on the perceived maintenance status of the property and yard’s shade cover. House and yard conditions were classified as follows: 1 = good maintenance, 2 = moderate maintenance, or 3 = no maintenance; shade coverage was classified in percentages: 1 = 0–25%, 2 = 26–50%, or 3 = 51–100%. Evaluations of house conditions were carried out by the same technician on each team before we entered the property, considering only what we saw from outside.

We classified inspected houses into two groups reflecting SELs using a two-step cluster analysis (TCA) in SPSS Statistics subscription (IBM Corporation, Armonk, NY). The algorithm creates subclusters following a hierarchical method and uses log-likelihood differences between subclusters as measures of similarity. On a second pass, Schwarz’s Bayesian Information Criterion (BIC) is produced for various or fixed (e.g., two clusters) solutions with different number of clusters, aiming at finding the largest distance between the more similar clusters at each stage in the hierarchical clustering. The optimal number of clusters is selected when the ratio of BIC values between more similar clusters is small. In our case, we ran the program with two fixed clusters to reflect contrasting SELs. All socioeconomic variables and house condition indices for households were entered as categorical variables.

We counted and classified every container that we found and recorded: the presence or absence of water, mosquito immature presence (larvae and pupae), and collected the pupae. Pupae were extracted by filtering the water into a tray using a sieve (2 mm diameter), rinsing and removing debris, and collecting pupae with droppers. Pupae were placed on damp paper towels in petri dishes and transported to the laboratory for identification.^17^ Emerging adult mosquitoes were sampled from septic tanks every day for four consecutive days using exit traps^18,19^; collected mosquitoes were transported to the laboratory and identified^20^; CDC, unpublished). We were not able to install exit traps in every septic tank because some residents did not allow it or because we were not going to have access to the traps in subsequent visits. In addition, we assessed the importance of roofs as possible *Ae. aegypti* production sites. We used a generalized linear model (GLM) to test for the effects of the following variables on the number of *Ae. aegy*pti pupae per property: status of the house (abandoned, vacant, or inhabited), cluster (low or medium SELs), and their interaction. We included weather variables as covariates in the analysis to account for possible significant effects because mosquito sampling was staggered in time. Mann–Whitney U tests (α = 0.05) were used to compare the medians of each of the House Condition Indices for the two SELs. We calculated Spearman correlation coefficients (α = 0.05) between house and yard maintenance indices for each SEL to understand if they were independent. Statistical analyses were conducted in SPSS Statistics subscription (IBM Corporation).

## RESULTS

We inspected 386 houses: 66 abandoned, 62 vacant, and 258 inhabited houses. The TCA classified the houses into two clusters with 47.7% and 52.3% of the houses. Variables contributing most to cluster separation were lot size, origin or developer of the neighborhoods, average family size, and percent of uninhabited houses. One cluster grouped houses with low SELs, which were initially established as invasions of public lands or land given by the municipality, and had larger lot sizes, smaller family size, lower property value, higher proportion of residents without complete high school education, lower percentage of houses built more than 30 years before, and lower income. The other cluster grouped houses with higher SELs. Indices of house condition did not contribute much to the separation of the clusters.

We identified 4,787 containers, of which 1,310 had water, 342 had immature mosquitoes, and 192 had mosquito pupae. The most abundant pupae were from *Culex* spp. (5,135), *Ae. aegypti* (1,632), *Aedes mediovitattus* (220), and other mosquitoes (33) (Table 1). Pupae of all mosquito species per house were more abundant in abandoned houses, followed by inhabited and vacant houses of low SELs.

**Table 1:** Abundance of containers with water and mosquito pupae in abandoned, inhabited, and vacant houses of two socioeconomic levels (low and medium) in southern Puerto Rico from May to August 2017

Cluster	House status	Inspected houses	Containers with water (containers per house)	Total *Aedes aegypti* pupae (pupae per house ± standard error)	Total *Aedes mediovitattus* pupae (pupae per house ± standard error)	Total *Culex* spp. pupae (pupae per house ± standard error)	Total other pupae (pupae per house ± standard error)
Low	Abandoned	36	175 (4.86 ± 1.25)	385 (10.69 ± 4.70)	68 (1.89 ± 1.73)	1,788 (49.67 ± 48.42)	0
	Inhabited	138	581 (4.21 ± 0.68)	1,057 (7.66 ± 2.14)	12 (0.09 ± 0.06)	3,199 (23.18 ± 19.67)	32 (0.23 ± 0.19)
	Vacant	32	39 (1.22 ± 0.28)	39 (1.22 ± 0.75)	6 (0.19 ± 0.19)	73 (2.28 ± 1.83)	0
Medium	Abandoned	30	29 (0.97 ± 0.23)	3 (0.10 ± 0.10)	0	18 (0.60 ± 0.60)	0
	Inhabited	120	453 (3.78 ± 0.38)	143 (1.19 ± 0.35)	134 (1.12 ± 1.12)	56 (0.47 ± 0.32)	1 (0.01 ± 0.01)
	Vacant	30	33 (1.10 ± 0.27)	5 (0.17 ± 0.08)	0	1 (0.03 ± 0.03)	0

The GLM comparing *Ae. aegypti* pupae per house was significant (likelihood ratio χ^2^ = 500.8; degrees of freedom [d.f.] = 8; *P* < 0.001), with significant effects of the status of the house (abandoned, vacant, or inhabited; Wald’s χ^2^ = 29.6; d.f. = 2; *P* < 0.001), cluster # (low and medium SELs; χ^2^ = 20.9; d.f. = 1; *P* < 0.001), and their interaction (χ^2^ = 20.7; d.f. = 2; *P* < 0.001). Accumulated rainfall (χ^2^ = 6.6; d.f. = 1; *P* < 0.05), temperature (χ^2^ = 25.7; d.f. = 1; *P* < 0.001), and relative humidity (χ^2^ = 11.6; d.f. = 1; *P* < 0.001) were significant covariates. Relative humidity was positively associated with the number of pupae per house, whereas rainfall and temperature were negatively associated. Pupal abundance of *Ae. aegypti* was higher in abandoned and inhabited houses of lower SELs (Table 1). The significant interaction term showed the uniformly low pupal abundance of *Ae. aegypti* in vacant houses, regardless of the SEL. Pupal abundance of other mosquito species, such as *Ae. mediovitattus* and *Culex* spp., was also higher in houses of low SELs (Table 1). Septic tanks were present only in low-SEL communities. Of 193 septic tanks, 51 were not sealed (cracks, broken pipes, vents without screen, or loose or broken caps), and of 32 septic tanks where we were able to use exit traps, we found that only nine were positive for *Ae. aegypti* adults: inhabited houses (5.9 ± 3.9 adults/house/day; *n* = 26), vacant (0.5 ± 0.5; *n* = 2), and abandoned houses (0.4 ± 0.2; *n* = 4).

**Figure 1.**
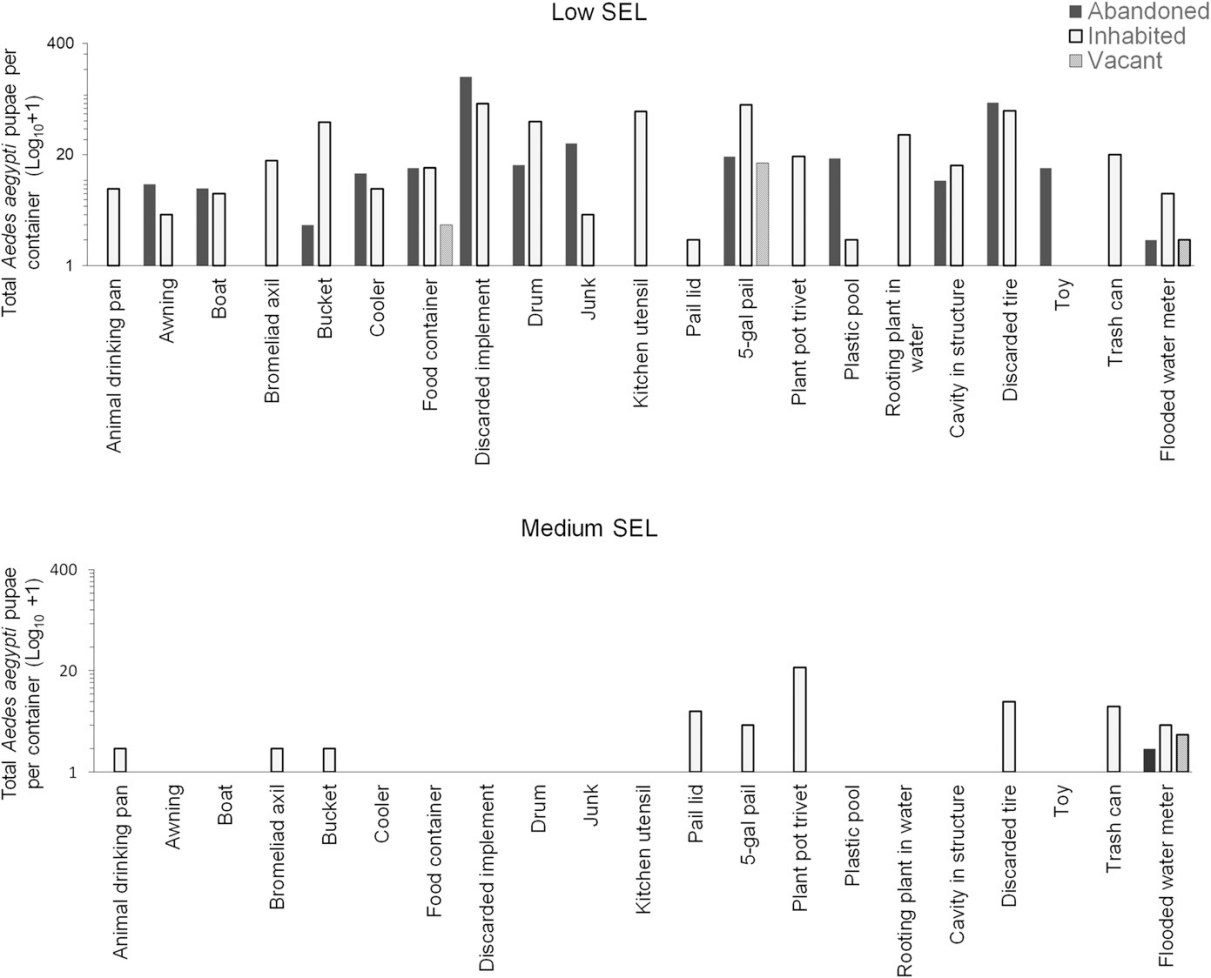
*Aedes aegypti* pupae per type of container found in abandoned, inhabited, and vacant houses of low and medium SELs in southern Puerto Rico. SEL = socioeconomic level.

Most of the containers with water (63.7%) and pupae of *Ae. aegypti* (93.1%) were found in low-SEL houses (Table 1). The lowest number of containers with water and mosquitoes was found in vacant houses in both SELs and in abandoned houses of medium SELs (Table 1). Few types of containers producing *Ae. aegypti* were found in vacant houses (discarded food containers, five-gal pails, and flooded water meters; Figure 1). There were 20 types of containers producing *Ae. aegypti* in inhabited houses of low SELs and only nine in medium SELs (Figure 1). There were 14 types of containers that produced most of the pupae in abandoned houses of low SELs and just one type in abandoned houses in medium SELs (flooded water meters; Figure 1). The percentage of disposable containers (trash) in abandoned houses was 90.1% and only 45.6% in inhabited houses of low SELs. The containers that we found in inhabited houses that were not observed in abandoned or vacant houses of low SELs were animal drinking pans, bromeliad axils, discarded kitchen utensils, rooting plants in water, plant pot trivets, and trash cans. These types of containers are related to daily human activities and reflect that houses were inhabited. The most productive containers in medium SELs were plant pots/trivets, tires, water meters, pail lids, and trash cans (Table 2, Figure 1). The most productive containers in low SELs were discarded implements (e.g., paint tray), tires, five-gal pails, discarded kitchen utensils, drums, buckets, and plant rooting in water (Table 2, Figure 1). The total number of pupae in houses of low and medium SELs was 1,481 and 151, respectively. Most pupae of *Ae. aegypti* (51%) were found in discarded containers in 24.1% of all containers, whereas 39.8% of the pupae were from useful containers that represented 36% of all containers in houses of low SELs.

**Table 2:** Pupae abundance per container type and its overall contribution (%) to the total number of pupae collected per SEL

Container	Low SEL			Medium SEL		
	*N*	Total number of pupae	%	*N*	Total number of pupae	%
Discarded implement	73	296	20	26	0	0
Tire	33	239	16.1	8	31	20.5
Five-gal pail	72	190	12.8	20	7	4.6
Kitchen utensil	28	154	10.4	8	0	0
Drum	14	119	8	16	7	4.6
Bucket	33	83	5.6	21	1	0.7
Rooting plant in water	29	66	4.5	3	0	0
Cavity in structure	15	47	3.2	12	0	0
Food container	106	40	2.7	21	0	0
Cooler	14	38	2.6	2	0	0
Plant pot/trivet	27	35	2.4	32	41	27.2
Junk	27	31	2.1	16	0	0
Boat	3	26	1.8	0	0	0
Bromeliad axil	51	22	1.5	68	2	1.3
Trash can	4	21	1.4	12	14	9.3
Plastic pool	6	18	1.2	5	0	0
Animal drinking pan	160	14	0.7	71	1	0.7
Toy	10	13	0.9	14	0	0
Water meter	30	13	0.9	84	29	19.2
Awning	12	11	1	12	0	0
Container not identified	2	3	0.2	0	0	0
Pail lid	22	2	0.1	56	18	11.9
Cistern	2	0	0	1	0	0
Coconut husk	10	0	0	1	0	0
Ornamental fountain	2	0	0	2	0	0
Bathtub	4	0	0	0	0	0

SEL = socioeconomic level.

Of 386 inspected houses, 258 had roofs that could retain water, but only 62 had standing water, and none was positive for immature mosquitoes. There were 52 roofs with water in medium-SEL communities versus 10 in low-SEL communities. We also found 68 water-holding containers on roofs; 17 of these had water, but none had immature mosquitoes. These containers were bottles, water tanks, pails, and tarps that some residents store on their roofs.

**Figure 2.**
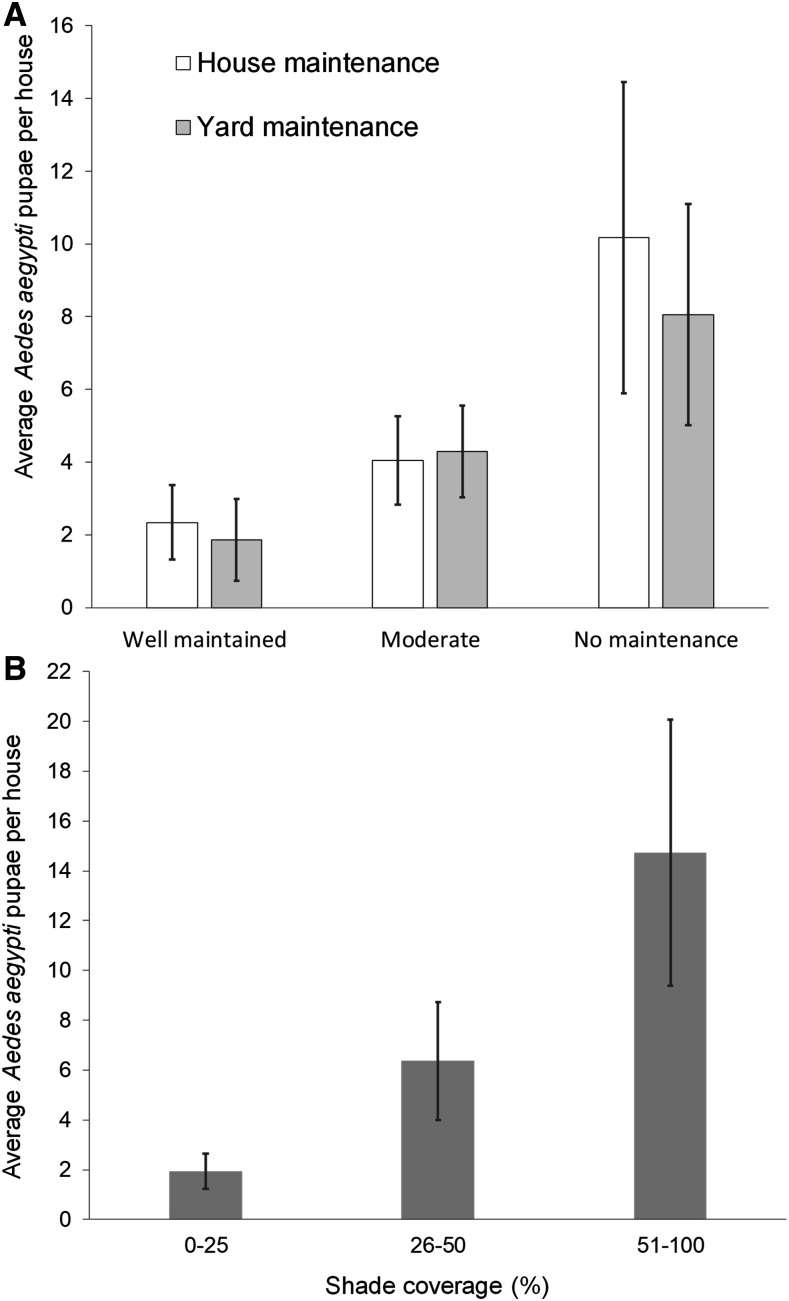
Average *Aedes aegypti* pupae per house by the House Condition Index (house maintenance, yard maintenance, and shade coverage) in 386 houses investigated in southern Puerto Rico.

We found more *Ae. aegypti* pupae in houses with poor house and yard maintenance and in houses with higher percentage of shade (Figure 2). Comparisons of median indices of house (*U* = 1.22; d.f. = 1; *P* > 0.05) and yard (*U* = 0.95; d.f. = 1; *P* > 0.05) maintenance between SELs were not significant. Indices of maintenance of the house and yard were significantly correlated in low (Spearman correlation index [rs] = 0.64; *P* < 001; *N* = 196) and medium (rs = 0.61; *P* < 001; *N* = 179) SELs. However, a comparison of median percentage of shade between SELs was significant (*U* = 21.7; d.f. = 1; *P* < 0.001). Houses of low SELs had higher percentages of yards with 50–100% shade (14.3%) than those in medium SELs (4.5%).

## DISCUSSION

The main aim of this study was to determine if vacant (for sale or rent, on travel) and abandoned houses were important contributors to *Ae. aegypti* populations in comparison with inhabited houses. This knowledge is important because traditional *Ae. aegypti* control approaches rely on inspecting houses and treating containers to reduce the population of this mosquito. A major difficulty with this approach is the usually low coverage or percentage of houses that inspectors can visit and treat because of residents’ absenteeism and refusals.^21^ If the percentage of uninhabited houses in neighborhoods is high, such as in the studied communities (24%), then it is more difficult to achieve significant mosquito control coverage. Studying the contribution of abandoned houses to *Ae. aegypti* productivity is also important because proximity to abandoned properties can be a risk factor for dengue infections.^22,23^

The results showed a significant interaction between the SEL and house occupancy, whereby abandoned and inhabited houses in low SELs produced the most *Ae. aegypti* pupae. Most of the containers found in abandoned houses of low SELs were discarded containers. By contrast, numbers of pupae in the medium SELs were significantly lower in abandoned, inhabited, and vacant houses. A lower number of pupae were found in vacant houses, regardless of the SEL. Therefore, despite low- and medium-SEL neighborhoods having similar number of unoccupied houses, abandoned properties in the medium SEL were not important producers of *Ae. aegypti* in this study. Abandoned houses in medium-SEL neighborhoods did not have an accumulation of discarded containers like that observed in low SELs. It would be important to understand why abandoned houses in medium SELs did not accumulate as many discarded containers. These results are useful to understand the spatial heterogeneity of *Ae. aegypti* and arbovirus transmission in urbanized areas in Puerto Rico. From the perspective of effective mosquito control, vector control agencies should have access to abandoned houses, which is often a difficult task. Our observations agree with several studies showing that people in low SELs are more exposed to mosquito bites and have a higher risk of dengue infections.^24–26^

The Premise Condition Index has been useful to rapidly identify houses with higher *Ae. aegypti* productivity based on the tidiness of the house and yard, and yard’s shade.^16,26^ We observed that the premise condition indices reflecting the tidiness of the house and yard did not differ between low and medium SELs. These two indices were significantly correlated within each SEL suggesting that both indices convey similar information. However, *Ae. aegypti* productivity was higher in houses with poorer house and yard maintenance, which is consistent with previous findings, and shows its potential value as a rapid indicator of mosquito production in this study. The diversity of types of containers with water and pupae of *Ae. aegypti* in medium SELs was lower and mainly made from containers in use by residents (e.g., animal drinking pans, plant pot trivets, trash cans, and bromeliads), whereas in low SELs, discarded containers predominated. We also observed that houses in low SELs had more vegetated, shaded yards and greater production of *Ae. aegypti* pupae, which is likely linked to the larger size of properties in houses of low SELs. Accumulation of discarded containers and number of trees on backyards were associated with greater productivity of *Ae. aegypti* in a previous study in Puerto Rico.^10^ Moreover, *Ae. aegypti* developing in containers under trees are larger.^26,27^ A more recent study on *Ae. albopictus* in Baltimore, MD, showed that this mosquito attains larger body size in blocks of lower SELs and greater level of abandonment.^28^

Another interesting finding was that septic tanks were found only in houses of low SELs. Although many septic tanks were cracked or open, the ones producing more *Ae. aegypti* adults were observed in inhabited houses. Lower production of *Ae. aegypti* in septic tanks of vacant and abandoned houses reflects their disuse, yet they still produced mosquitoes and should not be ignored by vector control programs. Open or broken septic tanks are important producers of *Ae. aegypti* and *Culex quinquefasciatus* in Puerto Rico, and mosquitoes emerging from septic tanks are larger than those emerging from surface containers.^18,29^ Larger mosquitoes have increased survival, fecundity, and frequency of blood feeding, which may increase their capacity to transmit pathogens.^28^ This investigation also included inspecting the roofs of houses, which is an exercise that is not frequently practiced, but we did not find any aquatic habitats producing *Ae. aegypti*.

One limitation of this study was that we were unable to sample houses in high SELs because when we were about to start, two major hurricanes hit Puerto Rico in September 2017. We observed that the landscape and house conditions after the hurricanes changed so much that a comparison with other SELs after hurricanes was not warranted. Hurricanes caused widespread damage to buildings, vegetation, roads, and power and telecommunications infrastructure in Puerto Rico. Spike increases in *Ae. aegypti* populations were observed five weeks after the initial impact, but mosquito abundance returned to pre-hurricane levels three months later.^30^ Limited circulation of arboviruses before the hurricanes was consistent with lack of circulation afterward, despite elevated *Ae. aegypti* populations.^30^ There was an estimated emigration of 160,000 people toward the mainland Unites States following hurricanes Irma and Maria that must have significantly increased the number of uninhabited houses in Puerto Rico.^31^

Another limitation was lack of information on the percentage of abandoned houses per neighborhood in each SEL. However, given the small numbers of containers producing pupae in abandoned houses of medium SELs, it is evident that the main concern for mosquito control purposes lies on abandoned and inhabited houses of low-SEL neighborhoods. The results from this investigation suggest that variations in SELs and their importance to *Ae. aegypti* productivity need to be explored further in Puerto Rico and elsewhere. If the results from this investigation can be extrapolated to other areas, we suggest that proper yard management could significantly reduce the production of *Ae. aegypti* and the risk of dengue infections in low-SEL neighborhoods.^32^

## Supplemental table and figure

Supplemental materials
