# Supplementary figures and images for: Role of Abandoned and Vacant Houses on Aedes aegypti Productivity
Source: Am J Trop Med Hyg. 2020 Oct 5;104(1):145–50. doi: 10.4269/ajtmh.20-0829 (PMC7790113; doi:10.4269/ajtmh.20-0829)

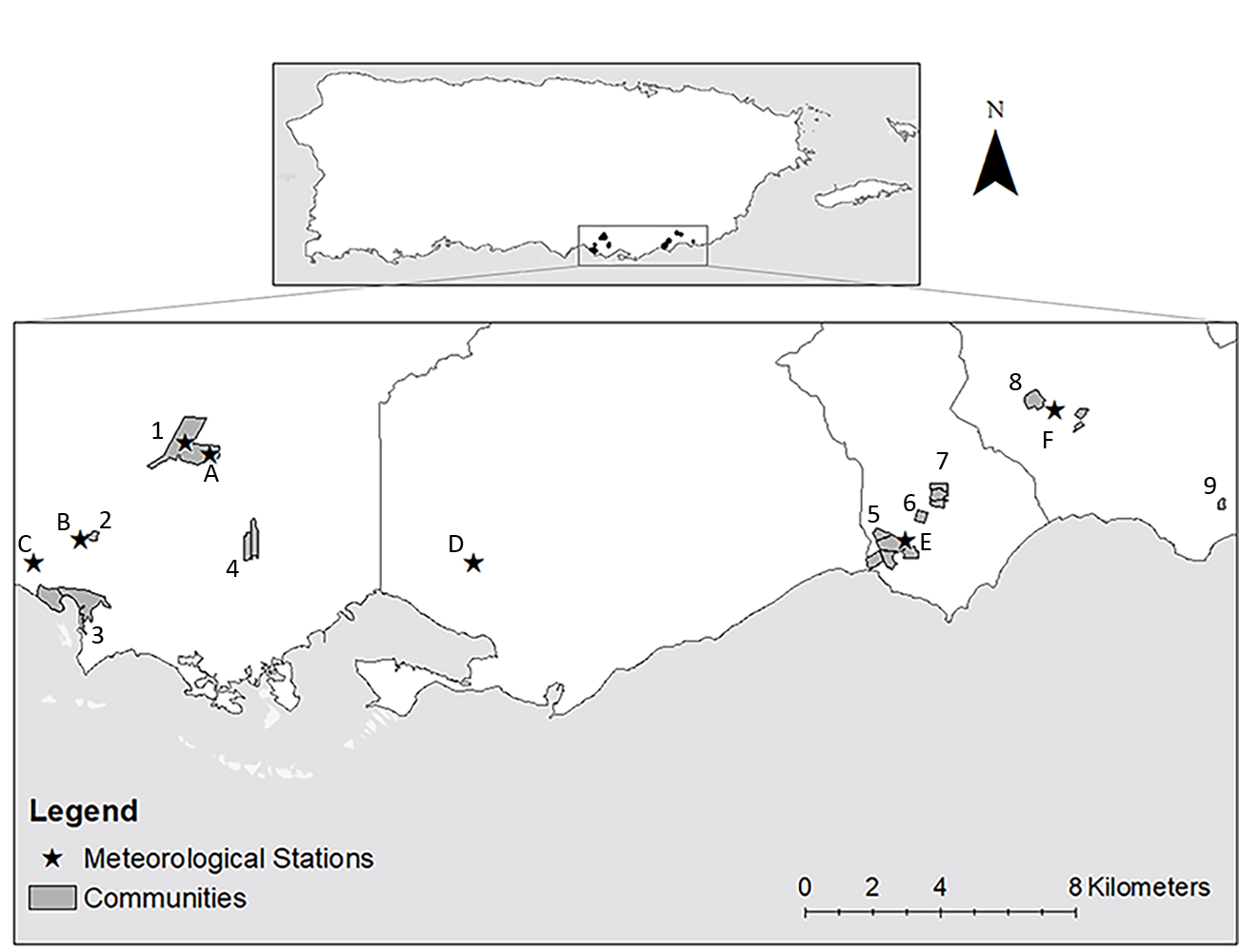

Supplement: Supplementary file 1 [file tpmd200829.SF1.tif]
