# Supplementary material for: Role of Abandoned and Vacant Houses on Aedes aegypti Productivity
Source: Am J Trop Med Hyg. 2020 Oct 5;104(1):145–50. doi: 10.4269/ajtmh.20-0829 (PMC7790113; doi:10.4269/ajtmh.20-0829)
Supplement: Supplementary file 2 [file tpmd200829.SD1.pdf]

Supplemental S1 Table. Location of studied communities and socio-economic variables.

Community	Municipality	Development of the neighborhoods[1]	Median Property value[2]	Median income[2]	Average family size[2]	% residents without complete high school education[2]	% residents over 60 years of age[2]	% uninhabited houses[2]	% older than 30 year[2]	Average lot size m[2 3]	Coordinates[4]	
Quintas de Guasima	Arroyo	Private sector	90300	19243	3.6	15.2	28.2	19.3	93.2	357	17° 58' 17" N	66° 03' 52" W
San Antonio	Arroyo	Private sector	85400	16130	3.5	23.8	28.2	36.7	86.6	344	17° 58' 11" N	66° 03' 31" W
Arroyo Village	Arroyo	Private sector	90500	17010	3.6	30.6	17.8	28.1	90.1	348	17° 59' 07" N	66° 03' 05" W
Belinda	Arroyo	Private sector	85400	16130	3.5	23.8	28.2	36.7	86.6	305	17° 58' 49" N	66° 03' 19" W
Jardines de Arroyo	Arroyo	Private sector	90300	19243	3.6	15.2	28.2	19.3	93.2	357	17° 58' 33" N	66° 04' 01" W
Park Guasima	Arroyo	Private sector	90300	19243	3.6	15.2	28.2	19.3	93.2	357	17° 58' 08" N	66° 04' 07" W
Valles de Arroyo	Arroyo	Private sector	90500	17010	3.6	30.6	17.8	28.1	90.1	348	17° 59' 11" N	66° 03' 02" W
Villas de Lafayette	Arroyo	Private sector	90300	19243	3.6	15.2	28.2	19.3	93.2	357	17° 58' 13" N	66° 03' 54" W
Vistas de Arroyo	Arroyo	Private sector	85400	16130	3.5	23.8	28.2	36.7	86.6	383	17° 59' 02" N	66° 03' 00" W
Jacaboa	Patillas	Private sector	89300	20606	3.6	35.6	30.1	34.1	92.6	267	17° 58' 56 " N	65° 58' 18" W
Marian	Patillas	Private sector	97800	17553	3.5	26.2	23.6	24.8	91.5	365	18° 00' 25" N	66° 00' 37" W
San Benito	Patillas	Private sector	97800	17553	3.5	26.2	23.6	24.8	91.5	365	18° 00' 13" N	66° 00' 40" W
Valles de Patillas	Patillas	Private sector	97800	17553	3.5	26.2	23.6	24.8	91.5	365	18° 00' 41" N	66° 01' 26" W
Paseo Costa del Sur	Salinas	Private sector	93300	27861	3.2	20.3	18.8	29.2	91.5	341	17° 58' 35" N	66° 14' 27" W
Coco	Salinas	Land provided by municipality	89800	18989	3.3	23.0	17.1	10.0	91.4	551	18° 00' 13" N	66° 15' 34" W
Jardines de Salinas	Salinas	Private sector	93300	27861	3.2	20.3	18.8	29.2	91.5	323	17° 58' 41" N	66° 17' 12" W
Playa	Salinas	Invasion	56200	12444	3.3	35.5	32.0	31.8	83.6	441	17° 57' 45" N	66° 17' 51" W
Playita	Salinas	Invasion	59300	17896	3.1	39.6	29.6	25.7	75.9	814	17° 57' 43" N	66° 17' 19" W
Trinitarias	Salinas	Private sector	93300	27861	3.2	20.3	18.8	29.2	91.5	341	17° 58' 33" N	66° 14' 33" W

[1] Data supplied my municipalities
[2] Data from US Census tracks
[3] Data calculated importing a layer of polygons describing lots supplied by the Property Tax Office of Puerto Rico into Geographic Information System software
[4] Coordinates of the center of communities acquired from Google Earth
