# Supplementary material for: Role of Abandoned and Vacant Houses on Aedes aegypti Productivity
Source: Am J Trop Med Hyg. 2020 Oct 5;104(1):145–50. doi: 10.4269/ajtmh.20-0829 (PMC7790113; doi:10.4269/ajtmh.20-0829)
Supplement: Supplementary file 3 [file tpmd200829.SD2.pdf]

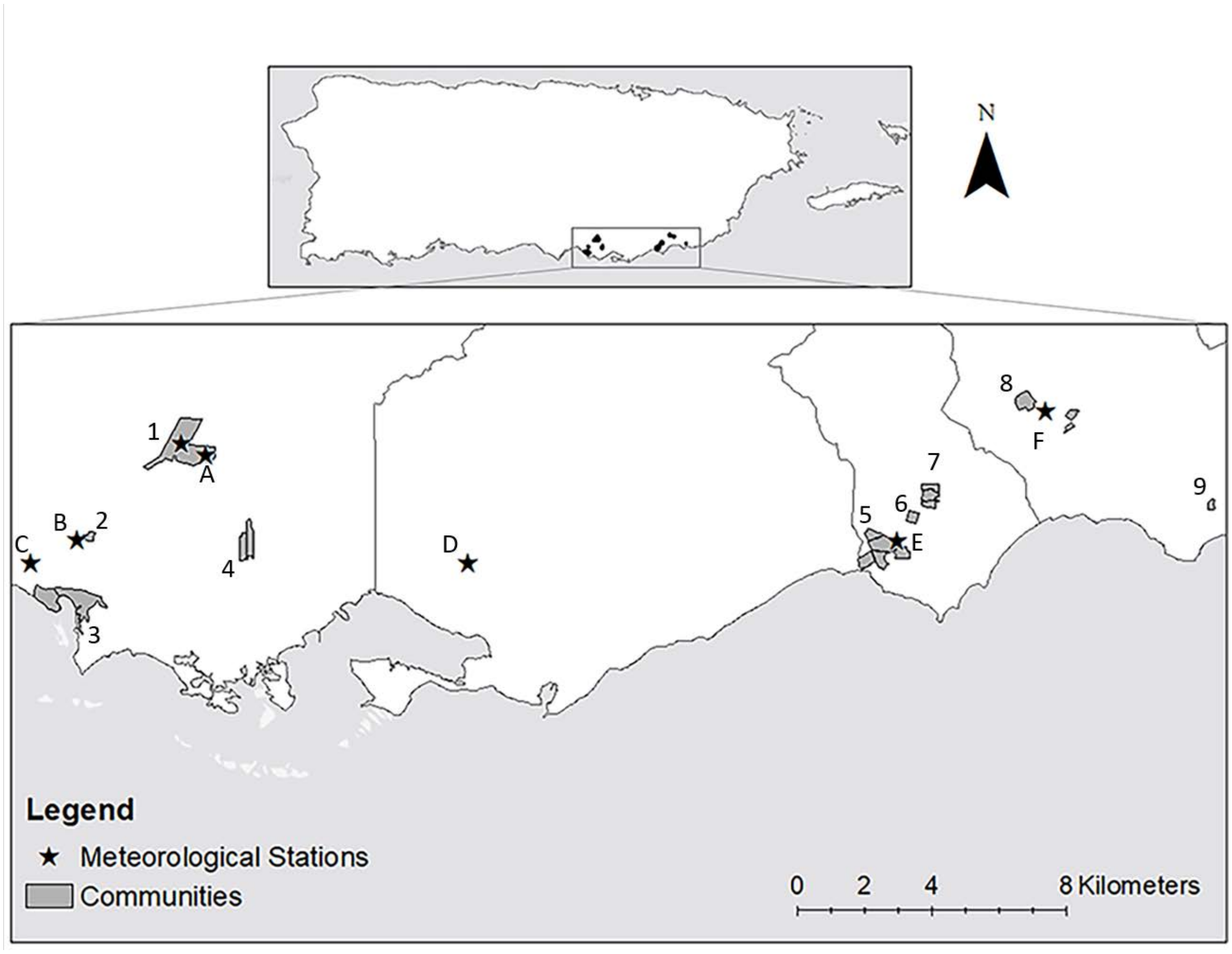

Supplemental S2 Figure. Map showing the location of the 19 neighborhoods that were studied in Salinas, Arroyo and Patillas municipalities, southern Puerto Rico from May to August 2017. Neighborhoods are indicated with numbers: 1- Coco, 2- Jardines de Salinas, 3- Playa, Playita, 4- Trinitarias, Paseo Costa del Sur, 5- San Antonio, Jardines de Arroyo, Jardines de Lafayette, Park Guásima, Quintas de Guasima, 6- Belinda, 7- Vistas de Arroyo, Arroyo Village, Valles de Arroyo, 8- Marian, San Benito, Valles de Patillas, and 9-Jacaboa. Location of meteorological stations are shown in uppercase letters: A- Coco, B- Arboleda, C- La Margarita, D- Villodas, E- Arroyo, and F- Patillas.
